# Supplementary material for: Development of New Diffuse Large B Cell Lymphoma Mouse Models
Source: Cancers (Basel). 2024 Aug 29;16(17):3006. doi: 10.3390/cancers16173006 (PMC11394112; doi:10.3390/cancers16173006)

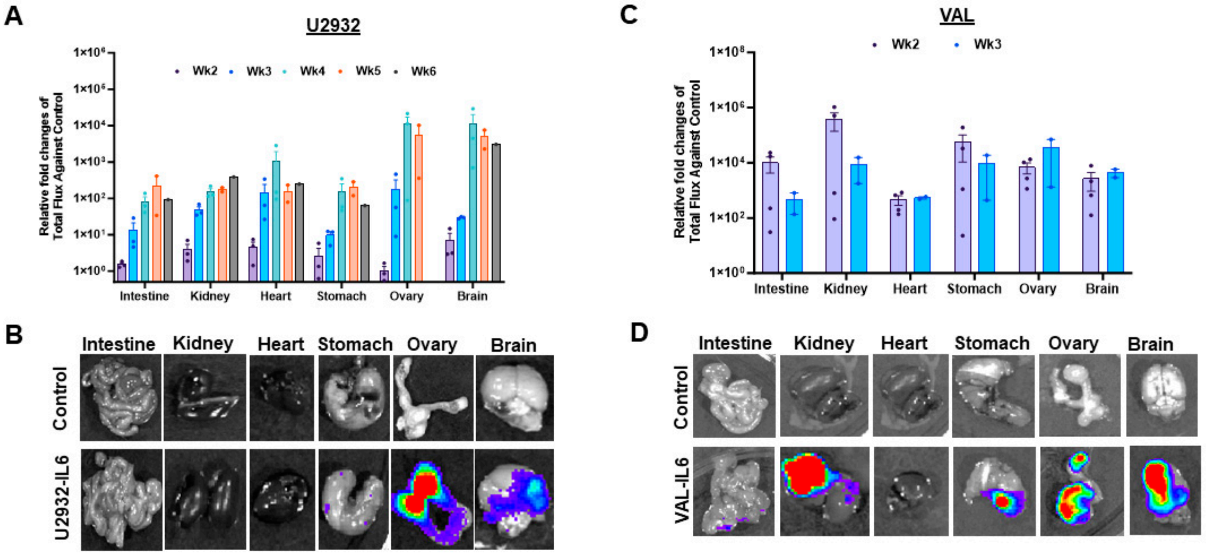

Figure S1. Total flux with ex vivo organ luminescence images of select organs from DLBCL intravenous xenografts. (A, C) Representative graphs show the ex vivo relative fold difference in organs regarding total flux against the control in U2932-Luc and VAL-Luc engrafted mice. (B, D) Organs from the U2932-Luc and VAL-Luc transfected mice were extracted and BL image was taken within 30 minutes after luciferin injection. All images were normalized for BL signals in the scale shown here.

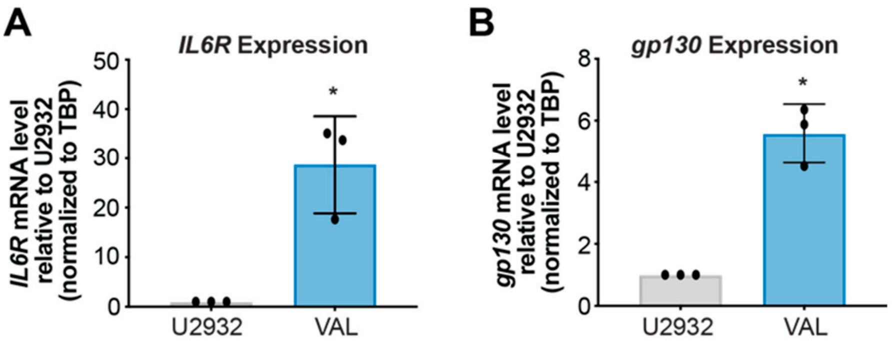

Figure S2. IL6R and gp130 mRNA expression in DLBCL cell lines. Basal mRNA levels of IL6R (A) or gp130 (B) in untreated U2932 and VAL DLBCL cell lines as detected by qPCR. Data represent mean ± s.d from three independent experiments. Adjusted p-values as determined by a two-tailed t-test with Welch's correction for significantly different variances. *$p < 0.05$.

Figure S3. Full pictures of the Western blots.

U2932 and VAL cells were treated in vitro with human IL6 (78148, StemCell Technol-ogy) at 50 ng/mL for 30 min and 60 min. For each lysate, 100 µg total protein was separated by SDS-PAGE (4 – 20% Mini-PROTEAN Precast Protein Gels, BIO-RAD) and transferred onto PVDF membranes. Membranes were probed with antibodies against TBP (ab818, Abcam; 1:2500), STAT3 (79D7, Cell Signaling Technology; 1:2000), and p-STAT3 (Tyr705, Cell Signaling Technology; 1:1000). Secondary antibodies used were goat anti-rabbit Dylight 800 (PISA535571, Thermofisher) and goat anti-mouse Dylight 650, (PISA510174, Thermofisher). Blots were imaged using the Biorad ChemiDoc MP (BIO-RAD) and images were analyzed using Image Lab software (BIO-RAD). Western blot analysis of STAT3 and phosphorylation of STAT3 (pSTAT3). STAT3 (A) and pSTAT3 (B) were detected at 88kD (Dyligh800). TBP (C)(D) was detected at 40kD (Dylight 650).

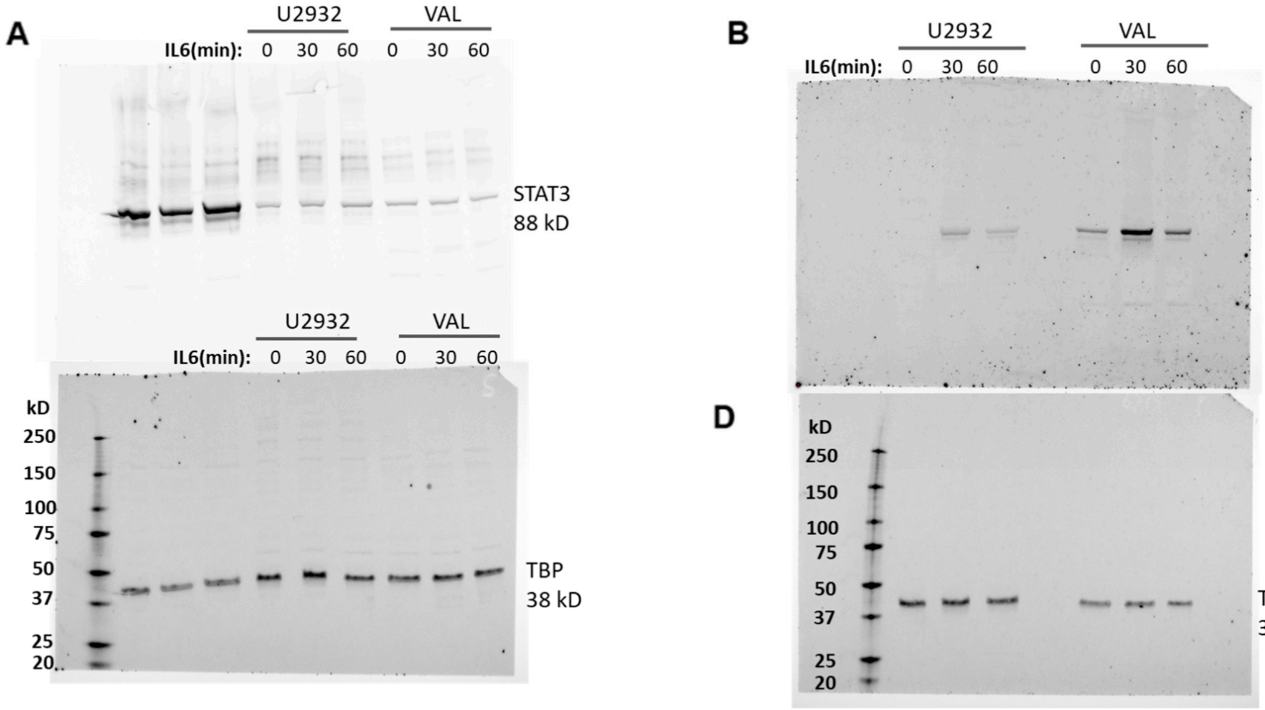

Supplement: Supplementary file 1 [file cancers-16-03006-s001.zip › cancers-3101734-supplementary.pdf]
